# A Synthesis of Findings from ‘Rapid Assessments’ of Disability and the COVID-19 Pandemic: Implications for Response and Disability-Inclusive Data Collection

**DOI:** 10.3390/ijerph18189701

**Published:** 2021-09-15

**Authors:** Tessa Hillgrove, Jen Blyth, Felix Kiefel-Johnson, Wesley Pryor

**Affiliations:** 1 CBM Inclusion Advisory Group, Box Hill, Melbourne, VIC 3128, Australia; jblyth@cbm.org.au; 2 Nossal Institute for Global Health, University of Melbourne, Parkville, Melbourne, VIC 3010, Australia; f.kiefeljohnson@unimelb.edu.au (F.K.-J.); wesley.pryor@unimelb.edu.au (W.P.)

**Keywords:** disability inclusion, inclusive development, COVID-19, disability data

## Abstract

Introduction: People with disabilities are disproportionately impacted by disasters, including health emergencies, and responses are not always inclusive or accessible. Disability-inclusive response and recovery efforts require rapid, contextually relevant data, but little was known about either the experience of people with disabilities in the first phase of the COVID-19 pandemic, or how rapid needs assessments were conducted. Methods: We reviewed the available results from rapid assessments of impacts of COVID-19 on people with disabilities in low- and middle-income countries in Asia and the Pacific. Rapid assessment methods and questions were examined to describe the current approaches and synthesise results. Results: Seventeen surveys met the inclusion criteria. The findings suggest that people with disabilities experienced less access to health, education, and social services and increased violence. The most rapid assessments were conducted by or with disabled person’s organisations (DPOs). The rapid assessment methods were varied, resulting in heterogeneous data between contexts. Efforts to standardise data collection in disability surveys are not reflected in practice. Conclusions: Persons with disabilities were disproportionately impacted by the ‘first wave’ of the COVID-19 pandemic. Despite complex implementation challenges and methodological limitations, persons with disabilities have led efforts to provide evidence to inform disability-inclusive pandemic responses.

## 1. Introduction

Nobody in the world has been unaffected by COVID-19, but people with disabilities are likely to be disproportionately affected by the pandemic’s health, economic, and social impacts. Research shows that people with disabilities are two to four times as likely to die or be injured in disasters, for example, during the 2011 earthquake in Japan [1] and Tropical Cyclone Pam in Vanuatu [1,2]. They are also more likely to be left behind in emergency responses and miss out on crucial humanitarian services due to a range of environmental, physical, and social barriers [3]. Global, national, and local efforts to mitigate COVID-19 infection risks such as lockdowns, social distancing, and quarantines have been highly varied, however, and are likely to disproportionately impact people with disabilities due to the interactions between impairments (e.g., mobility and communication) and barriers (such as inaccessible facilities and information), as well as difficulties preparing and recovering from disaster events [4]. Health and social systems to support persons with disabilities have been hindered by COVID-19 and mitigation strategies. The consequences are likely to be most acute in contexts where health and social systems are less responsive to specific requirements of people with disabilities. This includes low- and middle-income countries (LMICs), where it is estimated that 80% of the 1 billion people in the world who experience a disability live [5].

Since the onset of major COVID-19 mitigation measures in February 2020, many rapid needs assessments and surveys have informed the immediate responses, advocacy, and understanding of the experiences of people with disabilities. Disability-inclusive COVID-19 response and recovery efforts require robust evidence captured and made available quickly for response [6]. The recent development of consensus-based approaches to determine disability without a clinical assessment or self-identification such as the Washington Group Questions (WGQs) have led to advances in the comparability of experiences of people with and without disability and how disability is incorporated in early-response data collection efforts, but there is little information about how information about disability in health emergencies in LMICs is collected and used.

Rapid surveys conducted in the first year of the COVID-19 pandemic provided a time-critical opportunity to understand both the experiences of people with disabilities in the first phases of the pandemic and how disability-inclusive information is collected in rapid-onset public health emergencies. We aimed to examine both peer-reviewed and ‘grey literature’, recognising that peer-reviewed literature was likely to be scarce and focused on specific geographies and populations. Further, this review recognises ‘grey literature’ both as a valuable source of information [7] and as reflecting the evidence generated and used to inform humanitarian responses, especially in rapid, local responses. To contribute to the optimal use of information from rapid assessments conducted in Asia and the Pacific during the ‘first wave’, we had two aims: first, to synthesise the available evidence of the experiences of persons with disabilities, and, second, to report methodological approaches and their potential applications in future efforts to capture rapid information about disability in health emergencies, emphasising the potential role of local disability actors.

## 2. Materials and Methods

As part of our team’s role in providing technical advice for disability-inclusive development programming, we were requested to provide a rapid evidence summary of the emerging knowledge about COVID-19 among persons with disabilities in the Asia-Pacific region. The first phase of the review was undertaken between 17 May and 11 June 2020, with the second phase completed in January 2021 to capture the reports released in the second half of 2020. Our search strategy reflected the lack of peer-reviewed literature on disability and COVID-19 at the time and the limitations of conventional systematic review strategies in qualitative or mixed-methods research generally [8] and grey literature specifically [7]. The search strategy was informed by the ‘SPIDER’ (Sample, Phenomenon of Interest, Design, Evaluation, and Research type) tool. We thus explored broad terms using Google and Bing engines for the following elements: Persons with disabilities in the Asia-Pacific region (the sample), examples of consequences of COVID-19, and public health measures (Phenomenon of Interest). Our preliminary searches revealed highly varied approaches to the ‘first wave’ investigations and reports. We, therefore, adapted our inclusion criteria to allow for any Design, Evaluation and Research type. In this stage, we observed that disability was not explicitly defined in any report. We considered this both a useful finding and a challenge in synthesising findings and elected to include all the papers that satisfied the other criteria, regardless of the approach to defining disability.

Documents were hand-searched to identify additional reports or literature. We called for additional unpublished reports through our regional networks, including the Department of Foreign Affairs and Trade (DFAT)’s Disability, Indigenous and Social Inclusion Section and the Pacific Disability Forum. The search emphasised LMICs in East and South Asia, Southeast Asia, and Oceania (LMIC in line with the World Bank Atlas definitions) and global surveys where focal countries were included. All reporting identified was available in English. Using this approach, two researchers located 29 candidate documents. None were peer-reviewed. Twelve reports were excluded, because they did not focus on LMICs or did not report any primary data (such as advocacy papers and general guidance notes). Where questionnaires were available, they were screened to summarise the major themes of the questions, methodological features, and the organisations undertaking the survey.

## 3. Results

Table 1 summarises the reporting included in this. A total of 17 studies were included in the final review. Eleven were national studies: four from Indonesia, two from Cambodia, two from Nepal, one from the Philippines, one from Vietnam, and one from Bangladesh. Six global surveys were included, with one disaggregating findings to the regional level.

Overall, the findings point to concerning trends towards people with disabilities having been disproportionately affected by the COVID-19 pandemic. Despite limitations in the available data in this phase of the pandemic, the findings suggest poorer health access and outcomes, lower access to education, reduced services and supports, and increased violence and abuse among persons with disabilities. These themes are explored below.

### 3.1. Health

#### 3.1.1. Infection, Treatment, and Death from COVID-19

No available reporting provided disaggregated information about infection rates, access to treatment, or mortality among people with disabilities. There is patchy but concerning evidence of discriminatory triage policies and practices and inadequate medical treatment for COVID-19 among people with disabilities in institutional settings that directly or indirectly denied access to treatment [9]. Evidence about access to testing or rates of testing among persons with disabilities is limited, but findings from a global study noted access to free testing for people with disabilities in some Pacific Island Countries [10].

#### 3.1.2. Access to Public Health Information

Gaps in the accessibility of critical public health information, including information on preventing the transmission of COVID-19, where to seek testing and treatment, as well as restrictions on movements and changes in access to services, are apparent from the available findings. Self-reported knowledge related to COVID-19 (e.g., how to prevent infection) among respondents with disabilities was between 7% [11] and 50% [12].

Between one-third [11] and two-thirds [12] of respondents with disabilities in separate studies in Nepal reported health-related communications that were partly or not at all accessible to them, while an Indonesian report suggested 46% of respondents with disabilities found COVID-19 information ‘difficult to understand’ [13]. Barriers to inclusive health messaging include DPO leaders having inadequate information about COVID-19 and prevention measures against sharing information with people in their communities [12] and a lack of sign language interpreters for public information sharing and in health services [10]. The World Blind Union [14] reported the poor consideration of accessibility features for blind or partially sighted people in recommended ‘Covid Safe’ practices like contactless payments and physical distancing globally.

#### 3.1.3. Access to PPE and Infection Control Measures

Overall, the concern over the risk of infection was high (82%) among Vietnamese respondents with disabilities. This may be associated with low access to personal protective equipment (PPE) and inadequate soap and sanitisers to comply with health guidance, with 25% and 43% of respondents in Vietnam and Nepal, respectively, unable to access masks or sanitiser [12,15]. An Indonesia survey reported increased sanitation compared with the usual practices, with 75% of people with disabilities reporting an increase in infection prevention practices, but only 24% said that they practiced physical distancing, a proportion similar to the overall population sample [16]. Eighty-four percent of caregivers in a Nepal study reported using basic prevention measures, but the study authors reported a reduced likelihood of those measures among caregivers of women compared with caregivers of men [11], and overall, caregiver practices were not a common feature of the available studies.

#### 3.1.4. Access to Regular Health Care and Medicines

Difficulties accessing healthcare, including for check-ups, medicines, assistive devices, and rehabilitation, was experienced in every surveyed population (between 17 and 70% of respondents) [11,12,15]. A large global survey found that nearly all (96%) children with disabilities had reduced access to healthcare, medicine, and medical supplies during the pandemic, and six in 10 were unable to access their regular health and rehabilitation services [17]. In some instances, this was due to new physical distancing requirements, which prevented physical therapy [18]. In other settings, services related to pain management, sexual and reproductive health, breast cancer screening, and menopause were cancelled, postponed, or moved to telehealth formats that were not always accessible or appropriate for people with disabilities [19]. Disability-related services in health facilities appear to have been deprioritised as “non-emergencies”, and new barriers to accessing general health services emerged through a lack of transportation and new rules that did not allow support persons to accompany them [10]. There is evidence that, within general health services, the staff often did not wear masks and lacked protective equipment, putting service users at risk of infection [10].

Prices of medicines increased, and many individuals were forced to stop treatment for chronic conditions [9,10].

#### 3.1.5. Psychosocial Health

Six studies included questions addressing the experiences of psychosocial distress of people with disabilities; however, few explored the support needs, including of those with pre-existing psychosocial disabilities. Psychosocial distress of people with disabilities during the pandemic, especially when in lockdown, was reported by one in three people in a Nepal survey [11], nearly half of people in other surveys (Indonesia and global) [13,14], and up to 70% of respondents to a survey in Cambodia [20]. In a global study of people with vision impairment, people with existing mental health difficulties faced challenges accessing their regular support systems and mediation [14]. In another survey, 7% of all the respondents noted that psychological counselling was an immediate (unmet) support need [12].

### 3.2. Access to Regular Services and Supports

While definitions of disability services and supports vary between reports, consistent trends are apparent, with up to half of the respondents across the country reporting issues accessing their regular services and supports [9,11,13,19]. This has restricted people’s ability to live independently and increased their dependence on family members. In Nepal, 32% of those who needed personal support experienced reduced support or no support at all [11].

A global study outlined high proportions of people with disabilities globally who were unable to access essential services and support, such as personal assistance (38%), informal care (33%), and home support (29%), as well as assistive technology (23%). This placed further pressure on family support and/or left people with disabilities isolated and without the support required to enable them to live independently [9].

In several countries, personal assistants were not considered essential services by governments within the COVID-19 responses, leading to a decrease or cancellation of services [10].

### 3.3. Impact on Livelihoods

People with disabilities consistently reported a severe reduction in employment and income as a result of COVID-19 restrictions. In one comparative study, this was reported at a higher rate among people with disabilities compared with the general population [16]. Furthermore, as the surveys were undertaken in March and April 2020, at a time when the lockdowns were only newly introduced, these outcomes probably worsened as the lockdown continued.

In Indonesia, a large national survey found that 80% of people with disabilities who were active in the workforce before the pandemic experienced reduced incomes, often by a substantial amount (half of those with reductions lost 50–80% of their income) [21]. Other Indonesian surveys reported similar figures, including 84% in a second survey [13], and 67% of men and 71% of women (compared to 55% of men and women without disabilities) [16].

High proportions of income losses were also reported in Vietnam, which led to a considerable proportion of households falling into poverty for the first time (defined as a monthly income of below 1 million VND (or approx. $62 AUD)) [15]. In Nepal, one survey found that the restrictions negatively affected 76% of the respondents’ household incomes and 49% of their personal incomes [11], while a second survey found 40% of both men and women lost their incomes entirely and a further 20% were expecting to do so in the near future [12]. Households of people with disabilities in Cambodia and Bangladesh had lost more than half of their monthly incomes during the pandemic (52% and 65%, respectively) [20]. In Cambodia, losing a higher level of income was associated with being male (who, on average, had more income to lose) [20].

The shift to online platforms for work during the pandemic led to considerable accessibility barriers, compounded by low digital literacy, low confidence, limited access to information and communication technologies (ICTs), and required assistive technologies [14]. Internet connection was an issue across the Pacific, with households not able to access the bandwidth required to perform work activities from home. In some instances, people with disabilities reported employers using working from home measures to justify not fulfilling reasonable accommodation or accessibility requirements in the workplace (e.g., to use more accessible online platforms) [10]. Research in Cambodia highlighted that, for women entrepreneurs with disabilities, a lack of knowledge and skills in ICTs limited their ability to adapt their businesses in the face of the COVID-19 restrictions [22].

### 3.4. Access to Social Protection

Social protection programs were found to have substantial coverage gaps. For instance, a global survey of women, nonbinary, and trans people with disabilities demonstrated that people in informal work arrangements were particularly prone to missing out on social protection arrangements in their countries [19]. In Vietnam, substantial gaps in social protection coverage were reported for those in seasonal/informal jobs, informal business owners, and people with less severe forms of disability; only 13% of respondents received support in mid-April 2020. A low percentage of people accessing social protection coverage was also reported in Indonesia [21] and the Philippines [23]. 

Respondents also reported poor accessibility and inclusion in social protection schemes. For example, requirements to travel and present in person to receive financial support [15], a lack of accessible information about government support, an absence of disability as a targeting criterion in social protection programs [21], pre-existing barriers to setting up a bank account [10], and difficulties communicating with assessors for household benefits [23]. One study reported people who were deaf or hard of hearing were less likely to have accessed non-cash food assistance or conditional cash transfer coverage than people with other types of impairments [17]. 

One report described issues of cash assistance, where available, being insufficient for people with disabilities, as it covered only basic needs such as food and not the higher costs associated with disabilities, such as regular medication, specialised food, and personal hygiene supplies [23]. Cash assistance amounts also did not reflect the higher household expenses incurred during the pandemic for infection control and to increase internet access [18].

### 3.5. Food Security and Emergency Supplies

Food security during the COVID-19 pandemic was a common concern expressed by people with disabilities, with between one-third and three-quarters of respondents across most surveys noting this issue [9,10,11,12,15,23]. Reduced income, increased scarcity, the price of essential items, closing of marketplaces, and, in some locations, no access to transport were reported as reasons for skipping meals or reducing nutritional intakes. In some cases, respondents reported borrowing money or selling household goods to afford food [11]. Most households had limited or no stored food, and the majority of respondents in need were yet to receive food assistance. Limited access to social protection schemes and personal assistance added additional barriers to obtaining necessary food. Emergency food provision tended to be provided by NGOs and DPOs rather than the government and were more likely to be locally rather than nationally coordinated responses [9]. Like some social protection schemes, access to food and supplies often required attendance in person, which is an obvious barrier for many, especially people with disabilities [10].

A global survey focussing on disability rights also reported that children were among the most vulnerable groups of people with disabilities during the COVID-19 pandemic, facing reduced access to food, medicines, and support. While children living in poverty and in rural areas relied on NGOs for essential supplies, these did not always meet the needs of children with disabilities [9]. Services that previously provided assistance, such as school food programs, were unable to operate during lockdown restrictions, and in many countries, no alternative arrangements were put in place [24].

Beyond the need for food, disruptions in supply chains also reduced access to other essential items [10]. A Nepal survey found almost 40% of respondents needed sanitary and hygiene materials, such as sanitary pads, catheters, or incontinence pads, with emergency response organisations not meeting the full extent of their needs [11].

### 3.6. Education

At the time of this review, school closures affected around 90% of school children globally [17]. Evidence of education participation in home learning arrangements for children and young people with disabilities was not available in the review timeframe.

However, some reporting explored general trends for learners with disabilities. A needs assessment in Indonesia [18] reported a difficulty adapting to home learning among children with disabilities, compounded by limited access to electronic devices, especially when more than one child was at home. A global qualitative study found that respondents in Asia and the Pacific described challenges adapting to home learning that included general issues associated with taking on new duties supporting home learning, the increased cost of internet access, and difficulties with limited connections for those living outside urban centres [10]. The World Bank [24] reported that children with disabilities faced additional barriers of inaccessible learning platforms and educational content and limited access to the assistive devices (such as screen readers) that could address some of the barriers to remote learning, while poor support for parents with disabilities supporting home schooling arrangements is also an identified threat to education. Adults with blindness or low vision were also less able to shift to online learning [14]. Findings in Bangladesh described the delayed completion of degrees and entry into the job market among people with disabilities [25].

The World Bank report [24] highlighted how contexts with pre-existing “Universal Design for Learning” principles embedded have the potential to improve learning outcomes for children with disabilities during and beyond the pandemic. While not from the Asia-Pacific region, a good practice example was noted from Rwanda, where television lessons were accompanied by sign language interpretation, Braille scripts were developed and distributed to accompany radio lessons, accessible digital readers and textbooks were developed and disseminated, and guidance for parents to support remote learning for their children with disabilities was provided.

### 3.7. Experiences of Violence

There is strong evidence of grave human rights abuses against women and girls with disabilities, including sexual violence reported in both global [9] and country-level reports [25]. About a quarter of women, nonbinary, and trans people with disabilities in a global survey reporting increased fear of personal safety [19], while 40% of Cambodian respondents reported similar experiences or fears of violence [20].

Increased experiences of violence were variously attributed to a greater proximity to members of their household [19], power imbalances caused by increased dependence on others [19], and higher crime rates and stigma and discrimination from members of the public [10], which are typically known pre-existing risk factors but elevated during the pandemic. Support like police services, women’s shelters, social work services, or trauma counselling were disrupted or more difficult to access than usual, and people and children were often isolated at home with abusive partners and relatives without access to school or workplaces for reprieve [9]. In the worst situations, persons with disabilities reported police violence—including fatal violence—likely due to inaccessible information about curfews or other public health orders [10].

### 3.8. Survey Methods and Methodological Implications

#### 3.8.1. Sampling

Of the 17 surveys we examined, the sampling and recruitment approaches were highly varied. The sample (or sampling frame) was usually described as either people with disabilities and/or people who care for (families, professionals, etc.) people with disabilities. The samples were often described as purposive with no further description of what criteria were used to determine inclusion. The recruitment approaches also varied. Most used social media or word-of-mouth.

#### 3.8.2. Question Types and Survey Themes

The question types and survey themes were highly varied and context-specific. Few used accepted or consensus-based variables or question sets, such as the Washington Group Questions (WGQs). Disabilities were mostly determined using self-reported approaches or by sampling populations known to identify as experiencing disabilities. Self-reporting or self-identifying was the most common way of determining who experienced disabilities.

Consensus-based approaches like the WGQs were not prominent either as a screening approach for disability-specific surveys or a means to allow disability disaggregation of data. In disability-focused studies, other sociodemographic factors like age, gender, and living arrangements were highly varied, absent, or unclear. Questions often appeared to be worded poorly and/or to be quite complex for the expected respondents.

#### 3.8.3. Data Analysis

Of the quantitative studies, most emphasised descriptive statistics (proportions providing a response) but often without basic disaggregation (e.g., the results for men and women). Inferential analyses (for example, to determine if there were major differences between women and men, older/younger persons, and so on) were not common. Most surveys reporting differences for different population groups did not report whether the differences were statistically significant.

Only two studies compared the experiences of people with disabilities compared to the broader population. This analysis was able to highlight specific areas where people with disabilities had poorer outcomes in terms of livelihoods (Indonesia), and differences in accessing health services and medicines amongst children (global survey); however, this information is not available for all locations or thematic areas. Population surveys with disability-disaggregated data may be able to address this evidence gap, but none were available at the time of review.

#### 3.8.4. Other Methodological Features

Overall, obviously, due to the timing and specific aims of many of the rapid assessments we examined, very little information was available to participants about how survey data would be used, by whom, and where the findings might be reported. There was limited introductory information to help people understand whether to participate (such as length of the survey and whether contact information would be required), and only a few surveys used a formal ‘agree to participate’ option; none appeared to have formal approval from an ethical review board. At least one survey had the respondent details publicly available in the online survey software environment, highlighting some of the ethical risks observed overall.

## 4. Discussion

This analysis explored both (i) findings of studies exploring the impact of COVID-19 on people with disabilities and (ii) the implementation of data collection approaches. In the first phase of the pandemic, people with disabilities experienced disproportionate effects, mostly due to increased barriers to accessing information, health services, regular supports and services, livelihoods, and social protection measures.

The findings reported here were broadly consistent with the existing evidence demonstrating how the right to health, livelihood, education, and all other aspects of life is denied to many people with disabilities, especially in low- and middle-income countries pre-pandemic [26] and extended that knowledge to the unique context of the COVID-19 global pandemic, national preparedness, and responses. Our findings also highlighted the potential value but current challenges in conducting early assessments of the impacts of health emergencies generally.

The evidence from high-income countries shows that people with disabilities make up more than 50% of all COVID-19-related deaths [27]. An increased risk of death remains even after accounting for circumstances such as the place of residence (particularly group homes or institutions), socioeconomic and geographic factors, and age [28]. Given the disproportionate risk of illness and death, it is imperative that public health responses are accessible and inclusive and that people with disabilities are appropriately prioritised for preventative measures such as vaccinations.

Both the findings of the disproportionate effects on people with disabilities, and the methods used, highlight the importance of using approaches such as the WGQs in ‘mainstream’ surveys to illustrate the specific risk areas for people with disabilities overall and particular at-risk subpopulations. This gap in the evidence during the first wave of COVID-19 may have impacted the effectiveness and inclusiveness of the responses, further compounding the pre-existing exclusion and marginalisation.

These findings reveal that most of the effort and expertise used to give voice to the implications of COVID-19 on people with disabilities has been led by the disability community and civil society organisations. This is further evidence of the commitment to using data to inform responses and both the current and potential capacities of those organisations. There are clear opportunities to build on investments in data for disability-inclusive development actions and build a further capacity in regional stakeholders—especially DPOs—to be better prepared to survey their communities in future emergencies. The surveys explored here illustrated practical challenges in generating useful data to inform disability-inclusive policies and practices. These challenges are far greater than simply characterising disabilities through methods like the WGQs. Adapting new tools like the Coronavirus Disability Survey (COV-DIS) [29], developed to capture information about the impact of the COVID-19 pandemic on people with disabilities, can help improve the evidence for ongoing response and recovery during this emergency.

Disability-inclusive development is characterised by a focus on two main areas: (i) that the barriers to inclusion and opportunities for participation are identified and addressed, so people with disabilities can benefit on an equitable basis as others, and (ii) involving people with disabilities in planning, implementing, monitoring, and the evaluation of development programs. This review provides evidence that, in all the countries surveyed, people with disabilities experienced considerable barriers to accessing information, services, and support. These barriers need to be addressed as a matter of urgency in the ongoing COVID-19 response and recovery efforts, so people with disabilities are not left further behind in the development efforts. Crucially, future health emergency response and recovery efforts must involve people with disabilities and their representative organisations during planning and implementation. Research shows that people with disabilities are often not included in DRR activities or consulted when planning responses [29,30]. A number of organisations have developed guidelines to assist governments and other organisations in planning and undertaking preparedness and responses for COVID-19, to ensure that people with disabilities are considered in all aspects of outbreak mitigation and responses. These resources can be found on the “DID4all” COVID-19 & Disability Inclusion webpage (https://www.did4all.com.au/ accessed 30 August 2021) [31].

This rapid review has some important limitations. Most findings reflect the first months of the global COVID-19 pandemic. Both the infection and mortality rates were much higher in the subsequent months. In general, the public health measures became stricter later in 2020 and varied widely between countries and subnational regions. Our aim was to report broad trends and to critically examine the type and utility of the information collected in the first wave of rapid assessments. The summary findings are useful to highlight the general trends in the disproportionate effects of COVID-19 on people with disabilities. However, these trends should only provide general guidance on disability-inclusive programming and illustrate potential gaps in services. Due to the sampling approaches used, the results are unlikely to be statistically representative of the populations they are drawn from, and the point estimates (e.g., percentages experiencing a drop in income) summarised in this review should be interpreted with caution.

The timing and rapid nature of this review did not allow us to explore whether the survey findings were taken up in policy and practice. This would be an interesting area for future enquiries. The experience from previous emergencies and contexts suggests that, even when evidence demonstrates how people with disabilities are more at risk, there are political, budgetary, and social barriers to change. This is crucial in the context of pandemics, where policy responses are often very rapid and time-critical—there may be no opportunity for remedial strategies if those responses exclude persons with disabilities.

## 5. Conclusions

The findings from rapid reviews in the first year of the COVID-19 pandemic and associated public health measures reveal concerning trends about the experience of people with disabilities in LMIC in Asia and the Pacific. These impacts will result in serious setbacks in implementing the UN Convention on the Rights of Persons with Disabilities (CRPD) and achievement of the SDGs. Immediate action is required to ensure people with disabilities are not left further behind in the development efforts. This includes ensuring people with disabilities are appropriately prioritised for access to COVID-19 vaccinations.

The rapid assessments synthesised in this paper strongly indicate that people with disabilities have experienced considerable barriers to accessing government COVID-19 responses. People with disabilities are at risk of being left further behind in responses to subsequent waves of the pandemic and recovery efforts. Barriers to accessing the mainstream response efforts need to be addressed as a matter of urgency, including specific barriers faced by women and girls with disabilities. A key way to increase the inclusion and accessibility of the COVID-19 response and recovery efforts is to intentionally engage people with disabilities and their representative organisations in needs assessment, planning, implementation, and ongoing monitoring. 

The methods, samples, data collection, and analysis used in rapid reviews to date are highly varied, limiting the potential value of the data collection beyond its initial purpose. Despite the commitments and technical approaches for disability-inclusive information in assessments to inform emergency responses, robust evidence of the impact of COVID-19 on people with disabilities in the region is limited. DPOs and other CSOs responded to this gap by initiating disability-focused data collection, which could further be strengthened to provide crucial evidence during health emergencies.

## Figures and Tables

**Table 1:** Studies of disability and COVID-19 included in the evidence review.

Countries	Organisation	Timing of Data Collection	Sample Size (*n*)	Participants	Comments on Methods	Summary of Key Findings
Bangladesh (also Kenya, Nigeria, Uganda)	London School of Hygiene and Tropical Medicine [25]	July and August 2020	40 (in all 4 countries, Bangladesh total unclear)	Jobseekers with disabilities	Narrative interviews (no further detail available)	Respondents reported experiencing psychological distress due to stress, social isolation, and limited access to health services. Underemployment, financial insecurities and lack of social protection resulting in food, medicine and hygiene equipment scarcity. Setbacks in completion of degrees delayed entry into the job market. Increase in gender-based violence recorded in all countries.
Cambodia	Agile Development Group [22,25]	Unknown–before May 2020	19	Female entrepreneurs with disabilities	Telephone interviews.	Seventy percent of respondents reported anxiety and nearly half were experiencing depression. Limited knowledge and skills in ICT meant women entrepreneurs with disabilities had limited ability to adapt their businesses to prosper under COVID-19 restrictions.
Cambodia	ADD International (funded by DFAT) [20]	July–September 2020	87 members, 10 leaders	Disabled Person’s Organisations (DPO) members (80% female); DPO/Self-help group leaders	Telephone interviews. Purposive sampling.	Fifty-two percent of respondents reported losing more than half their monthly income during the pandemic. This disproportionately affected women and people with communication and self-care difficulties. Most respondents reported having a shortage of food. Respondents reported difficulties accessing pandemic supports on an equal basis to people without disabilities. Forty percent of respondents reported increased risk of violence, with economic distress a contributing factor. Up to 70% of respondents reported experiencing psychosocial distress.
Indonesia	Arbeiter-Samariter-Bund (ASB), and a consortium of disability organisations [13]	24–29 March 2020	221	Both people with and without disabilities (32% had a disability)	Online survey, quantitative	Almost half of respondents reported COVID-19 information as difficult to understand. Nearly half experienced reductions in daily activity supports. Eighty percent of respondents experienced significant reductions in income. Almost half of respondents reported experiencing psychological distress.
Indonesia	Harvard, MIT, and J-PAL SEA [16]	13–15 April 2020	205	People with disabilities	Online survey, quantitative. One of the few surveys that compares results to a mainstream population, to note where the experience of people with disabilities differs from others in the population.	One in four respondents reported an increase in personal protective practices during the pandemic. In line with the broader population, 24% said they practiced social distancing. People with disabilities experienced higher rates of income and employment loss than people without disabilities, in addition to gaps in social protection. Up to three quarters of households experienced food insecurity.
Indonesia	YAKKUM (health service provider) [18]	Early April 2020	92	People with psychosocial disabilities (59), people with other impairments (15), parents of children with cerebral palsy (17)	Not described	People with disabilities experienced significant barriers to accessing information. Deaf people reported challenges communicating due to mask wearing. Mainstream social media as well as social networks were the most common sources of COVID-19 information. Children with cerebral palsy experienced interruptions in therapeutic supports due to social distancing measures. Job losses and reduced household income were common with limited access to social protection. Respondents reported increased psychological distress and expressed fear of using health services and public amenities due to risk of infection. Children with disabilities found to have difficulties accessing and participating in remote learning activities.
Indonesia	DPO Network in Indonesia (70 DPOs in total) [21]	10–24 April 2020	1683	People with disabilities	Online survey, quantitative	Most respondents reported reductions in employment and income. Ineligibility and lack of accessible information caused gaps in social protection coverage.
Nepal	National Federation of the Disabled Nepal (umbrella body for 331 member organisations throughout the country) [12]	12–22 April 2020	422 people with disabilities, 101 DPO leaders.	People with disabilities, DPO leaders	Telephone interview, quantitative. Purposive sampling possibly used. Sign-language interpretation available for deaf participants.	Gaps in knowledge reported around lockdown measures and how to prevent COVID-19 transmission. People with significant disabilities experienced greater barriers to accessing COVID-19 information. DPO leaders reported limited means of sharing information with people with disabilities. Nearly half of respondents reported limited access to PPE. Psychological distress and need for mental health supports were common during the pandemic. Few government isolation and quarantine facilities deemed to be accessible.
Nepal	Humanity and Inclusion (INGO) [11]	5–8 April 2020	686	People with disabilities	Phone interview (incl proxy), quantitative	Gaps in knowledge around how to prevent COVID-19 transmission. Low understanding of COVID-10 protection and safety messages. People with significant disabilities had more difficulties accessing COVID-19 information. Almost a third of respondents had reduced access to usual services and supports. Twenty-seven percent of respondents reported interrupted access to medical and assistive services. Carers of women with disabilities were less likely to follow COVID-19 preventative measures than carers of men with disabilities. Seventy-six percent of respondents’ incomes were negatively affected, with 40 percent losing their income entirely. Four-in-ten reported food insecurity and most were unaware of food relief packages.
Philippines	Centre for Disease Preparedness (NGO, that works with DPOs) [23]	28 April to 2 May 2020	1313	Persons with disabilities and without disabilities	Online survey, methods not clear in report	Loss of income led to unfulfilled basic needs, the foregoing of therapies, and an inability to purchase medication and assistive products. Most respondents were unable to access social protection and promised welfare benefits.
Vietnam	UNDP, with support from DFAT, the Embassy of Ireland in Viet Nam and the Korea International Cooperation Agency (KOICA) [15]	14–28 April 2020	986	People with disabilities	Online survey, phone and face-to-face methods to maximise participation in rural/remote areas; quantitative	Seventy percent of respondents reported difficulties accessing healthcare. Respondents reported difficulties accessing PPE and expressed concern around being able to protect their own health during the pandemic. Thirty percent of respondents became unemployed and 28% experienced loss of income, with some falling below the poverty line. Gaps in social protection for people with disabilities, particularly those in informal and seasonal employment. Despite increased reliance on food and financial assistance, only 16 percent had received these.
Global (data disaggregated for Asia and the Pacific)	Stakeholder Group of Persons with Disabilities for Sustainable Development [10]	1 May to 5 June 2020	106 (28 from Asia and Pacific)	People with disabilities	Online interviews and focus group webinars; qualitative. Interviews conducted in six languages, and featured online captioning, and International Sign interpretation.	Rising cost of health services and medicine forced people with chronic health conditions to stop treatment. New triage policies and practices denied individuals access to medical treatment. Disability services in health facilities were deprioritised. In the Asia Pacific region, respondents reported decreases and cancellation of personal assistant services and higher reliance on informal supports. Minimal cases of COVID-19 and access to free testing reported in Pacific Islands countries. Some examples of inclusive health messaging but not in all countries, with limited access to sign language interpreters a major barrier. Many could not access financial supports and welfare benefits, in some cases due to falling outside eligibility criteria. Accessible information, disruptions in transportation, and communication barriers limited respondents’ access to PPE. Respondents reported increased violence from police. Reports of facilities and institutions closing suddenly, leaving some residents without care.
Global	Seven disability organisations [9]	April–August 2020	2152	People with disabilities; representatives from DPOs; policy makers;	Online survey with print versions available; Available in 25 different languages. Questions focus on policy responses at the national level. Lower participation from Asia and the Pacific	People living in institutions were not receiving adequate medical treatment for COVID-19. People with disabilities rely more on families for support due to limited access to regular services and supports. One third of respondents reported being unable to access food. Limited access to social services and more time spent at home caused increased family and domestic violence.
Global	Women Enabled International [19]	March and April 2020	100	Women, nonbinary (typically defined as a gender identities that are neither male nor female—outside the gender binary), trans, and gender non-confirming persons with disabilities	Online survey, qualitative. Respondents primarily from North America, little representation from Asia and the Pacific	Restrictions on movement a factor in one-in-three respondents losing access to usual disability supports and services as well as informal support from family and friends. Respondents experienced increased psychological distress and overall reductions in access to health services and assistive technology. Most respondents feared people with disabilities would be deprioritised in the rationing of healthcare. Fifty-seven percent of respondents experienced reductions in employment and income.
Global	World Bank’s Inclusive Education Initiative (IEI) [24]	12 March 2020 to 24 May 2020	3993	Parents/caregivers of children with disabilities, teachers of children with disabilities, and persons with disabilities	Online survey, quantitative with open-ended response questions. Survey available in 5 languages.	Children with disabilities identified as particularly vulnerable to food scarcity and reduced access to medicine and disability supports. Humanitarian food distribution in rural areas were inadequate in meeting the needs of children with disabilities. Shift to remote learning disproportionately impacted on students with disabilities due to reliance on digital technologies.
Global	World Blind Union [14]	Unknown. Published in August 2020	853	People who are blind or partially sighted	Online survey, quantitative and open-ended questions; available in three languages.	New COVID-19 hygiene measures including PPE did not account for needs of blind or partially sighted people. Respondents reported increases in anxiety and depression. Top concerns of respondents during the pandemic were transportation and mobility as well as maintaining independence, autonomy, and dignity. Adults and children with disabilities alike experienced difficulties accessing and participating in remote learning and online work. Social and physical distancing regulations made accessing public amenities and performing community activities of daily living more difficult.
Global	Save the Children [17]	Unknown. Published September 2020	17,565 parents and caregivers, 8069 children	Save the Children programme participants across 37 countries. % with disabilities not reported	Online survey, quantitative and qualitative. Limited reporting of the experiences of children with disabilities.	Children with disabilities had reduced access to healthcare, including medicine, as well as social protection during the pandemic. Report finds specific needs of children with disabilities are not reflected in national policies and practices around poverty reduction, and food and shelter provision. School closures are predicted to exacerbate barriers to learning.
